# Antisense oligonucleotide-mediated disruption of mRNA localisation inhibits angiogenesis

**DOI:** 10.1007/s10456-026-10087-z

**Published:** 2026-08-20

**Authors:** Josy Augustine, Precious O. Owuamalam, Jennifer Nicell, Peter Barabas, Susanta Chatterjee, Michael O’Hare, Tim M. Curtis, Guilherme Costa

**Affiliations:** 1 Wellcome-Wolfson Institute for Experimental Medicine, Queen’s University, Belfast, BT9 7BL UK; 2 Johnston Cancer Research Centre, Queen’s University, Belfast, BT9 7AE UK

**Keywords:** Antisense oligonucleotides, mRNA localisation, Endothelial cells, Angiogenic sprouting

## Abstract

mRNA localisation is a critical posttranscriptional mechanism that confers a spatiotemporal dimension to the control of gene expression. Among diverse outcomes, this process can result in compartmentalised protein synthesis and consequently, elicit localised cellular responses. Targeting mRNAs to their destination is often determined by localisation elements (LEs) contained in untranslated regions within targeted transcripts. Although mRNA localisation has been widely explored in the context of subcellular biology, its roles in tissue function are only just beginning to emerge. A defined set of transcripts accumulate at the leading edge of endothelial tip cells that guide emerging vessels during sprouting angiogenesis. This includes *RAB13* and *NET1* mRNAs, which encode proteins implicated in cytoskeletal remodelling processes underpinning cell motility. In this study, we tested the anti-angiogenic potential of antisense oligonucleotide (ASO)-based strategies designed to perturb *RAB13* and *NET1* localisation. Upon confirming that ASOs targeting LEs mislocalise these mRNAs without altering steady-state levels of the encoded proteins, we applied them to a series of in vitro, ex vivo and in vivo angiogenesis assays. Remarkably, the mislocalisation of *RAB13* and *NET1* inhibits chemotaxis and vessel sprouting in response to pro-angiogenic stimuli. Furthermore, vessel sprouting from mouse choroidal explants and retinal angiogenesis are also hindered by mRNA mislocalisation. Altogether, our strategy for disrupting spatial control of gene expression in endothelial cells opens new mechanistic avenues for the manipulation of vessel formation.

**Supplementary Information:**

The online version contains supplementary material available at 10.1007/s10456-026-10087-z.

## Introduction

Subcellular mRNA localisation and subsequent compartmentalised protein synthesis are determinant mechanisms of spatial control of gene expression. Local translation of mRNAs is understood to generate in situ pools of “new” proteins intrinsically distinct from “older” versions. This may result in intracellular protein asymmetries critical for fine-tuning cell behaviour and morphology [1, 2]. In the context of cell migration and adhesion, accumulation of key mRNAs at the leading-edge of motile cells contributes to the regulation of cell shape and movement. mRNA localisation is mediated by cis-regulatory elements, or localisation elements (LEs), which are frequently present within their 3′ untranslated regions (3′ UTRs) [3]. LEs contain targeting information through defined nucleotide sequences and secondary structures recognised by the cellular machinery responsible for active mRNA transport and subcellular compartmentalisation [4].

Our prior work implicated mRNA localisation in sprouting angiogenesis [5], an intricate morphogenetic process in which highly motile *tip* endothelial cells lead the front of branching vessels, followed by trailing endothelial cells that form new vascular networks [6]. Like other motile cells, *tip* cells display asymmetrical distribution of mRNAs along the axis of migration [5, 7]. A characteristic subset of transcripts accumulates at the leading-edge of *tip* cells, some of which encode cytoskeletal modulators with the capacity to shape the cell front during movement. Among the latter, *RAB13* and *NET1* have been the focus of several studies that dissected mechanisms and roles of mRNA localisation in endothelial cells and beyond [5, 8–10]. The distinct distribution of *RAB13* and *NET1* is driven by 3′ UTR LEs composed of GA-rich sequences [5, 10]. We have shown that genomic excision of LEs in *tip* endothelial cells results in loss of mRNA localisation and altered morphological polarity [5]. Hence, disruption of mRNA localisation to reduce functional protein activity solely where translation takes place is an alternative route to traditional protein ablation for the perturbation of angiogenesis. While our previous approach relied on cumbersome genetic manipulation tools, others have used antisense oligonucleotides (ASOs) targeting LEs in metastatic breast cancer cells and reported reduced collective invasion in vitro [11]. Given the practical advantages of direct mRNA targeting, ASO-based strategies represent an attractive approach to further interrogate the roles of *RAB13* and *NET1* in angiogenic sprouting and, potentially, to develop novel drugs to curb undesired angiogenesis.

ASOs target RNA via complementary base-pairing and, depending on chemical modifications, can act through diverse posttranscriptional mechanisms to regulate gene expression. Such versatility has been key in the development of ASO-based drugs, which now represent an established therapeutic modality targeting a spectrum of diseases [12]. Stagewise advances in ASO chemistry, particularly backbone and sugar modifications, have significantly improved efficacy and underpinned successful drug development. First-generation ASOs, typically containing phosphorothioate (PS) backbone modifications [13], showed enhanced nuclease resistance but were limited by nonspecific protein binding and off-target effects. Second-generation strategies introduced ribose modifications such as 2′-O-methyl (2′-OMe) and 2′-O-methoxyethyl (2′-MOE), improving binding affinity, stability, and biocompatibility [14]. More recent chemistries such as locked nucleic acids [15] and phosphorodiamidate morpholino oligomers (PMOs) [16], provide further gains in stability and affinity. Nevertheless, due to increased manufacturing costs, challenges in intracellular delivery, and hepatic toxicity associated with some third-generation ASOs, second-generation chemistries are often the preferred choice [12].

Here, we compared the efficacy of 2′-MOE ASOs designed to mislocalise either *RAB13* or *NET1* mRNAs in the regulation of angiogenesis. We first demonstrate that ASOs disrupt mRNA localisation in primary human endothelial cells, without affecting steady-state RNA and protein levels. Next, we show that mislocalisation ASOs disrupt chemotaxis-induced endothelial cell migration and reduce angiogenic sprouting, with varying potencies, in advanced in vitro systems. Subsequently, we examined whether abnormal vessel formation could be recapitulated in an ex vivo murine model of tissue angiogenesis with ASOs targeting *RAB13* and *NET1* orthologues. Finally, we validated our findings in vivo through intravitreal administration of mislocalisation ASOs into mouse eyes and demonstrate their distinct efficacies in suppressing angiogenesis in a revascularisation model. Altogether, our findings provide new strategies to manipulate angiogenesis via spatial control of gene expression, paving the way for targeted approaches to manage pathological neovascularisation.

## Results

### ASOs targeting LEs disrupt mRNA localisation in endothelial cells

**Fig. 1:**
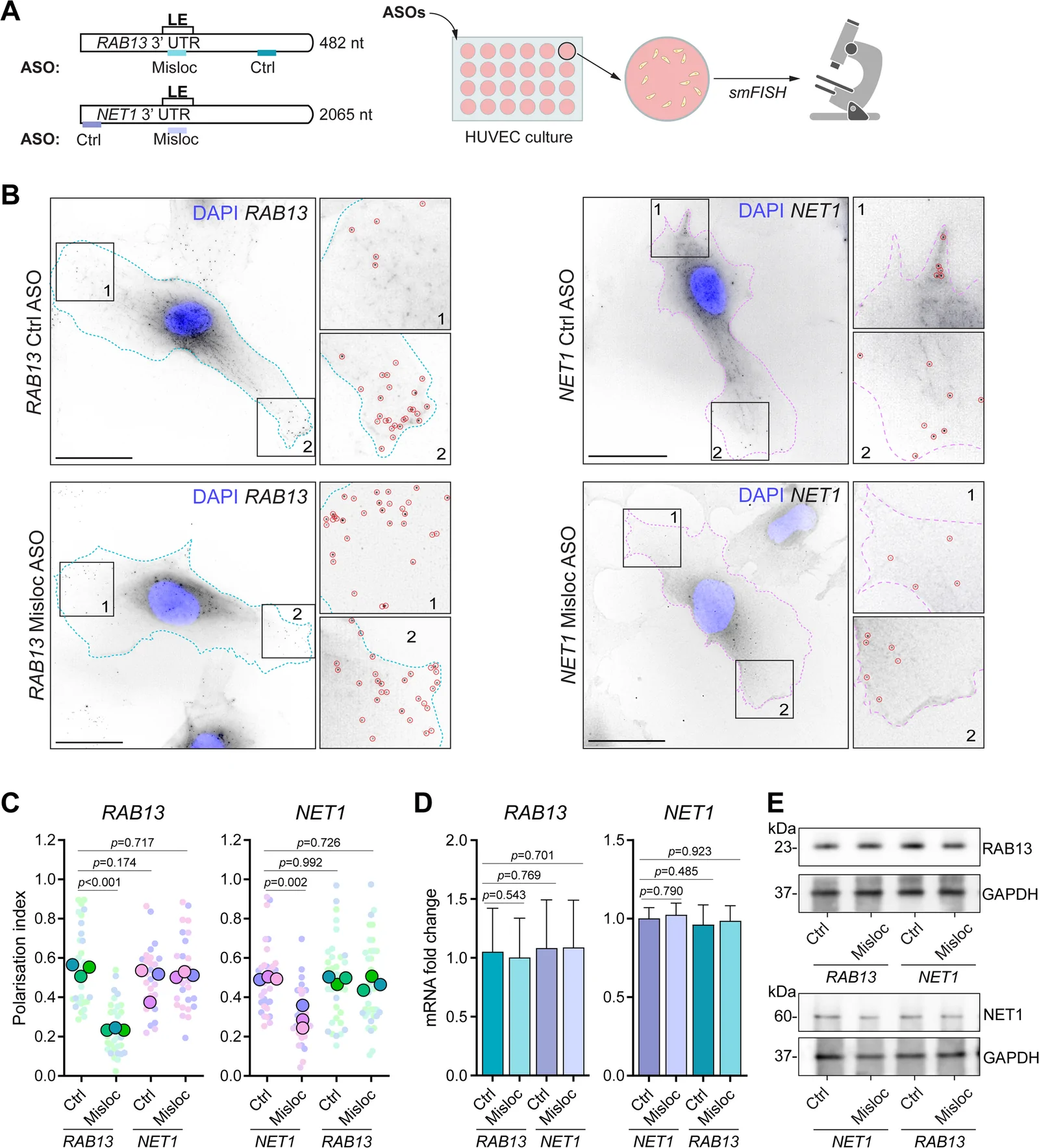
2′-MOE-PS ASOs targeting LEs disrupt mRNA localisation in HUVECs. **A** Diagram depicts 3′ UTR positions targeted by *RAB13* and *NET1* ASOs (left), and the strategy used to assess their mRNA mislocalisation potential in HUVECs via smFISH (right). **B** Representative *RAB13* and *NET1* smFISH images; insets illustrate the relative abundance of smFISH puncta at each cell pole; scale bars: 50 µm. **C** Scatter plots show the polarisation index of *RAB13* and *NET1* mRNAs; n = 3 biological replicates, > 7 cells per replicate; one-way ANOVA followed by Dunnett’s multiple comparisons test. **D** Bar graphs represent RT-qPCR quantification of *RAB13* and *NET1* mRNA in HUVECs; n = 3 biological replicates; means ± standard deviation; one-way ANOVA followed by Dunnett’s multiple comparisons test. **E** Representative blots of RAB13 and NET1 levels in HUVECs

*RAB13* and *NET1* mRNAs accumulate in protrusions at the leading edge of motile endothelial cells. Previous studies, including our own, have identified GA-rich LEs within their 3′ UTRs that are critical for their characteristic polarised distribution [5, 10]. To disturb mRNA localisation with antisense strategies, we designed 25 nucleotide-long 2′-MOE-PS ASOs complementary to *RAB13* and *NET1* LEs (misloc ASOs), and control counterparts (ctrl ASOs) targeting 3′ UTRs regions with no apparent localisation properties (Table S1). Human umbilical vein endothelial cells (HUVECs) and human retinal microvascular retinal cells (HRMECs) were cultured with ASO-containing media, and subcellular mRNA distribution was analysed (Figs. 1A, S1A). We employed single-molecule fluorescence in situ hybridisation (smFISH), which enables visualisation of individual mRNA molecules. While cells cultured with ctrl ASOs retained the typical polarised distribution of *RAB13* and *NET1* mRNAs, uptake of misloc ASOs disrupted the localisation of their respective targets (Figs. 1B, S1B).

To quantify this effect, we calculated mRNA polarisation index (PI), a measure of asymmetrical localisation determined by dividing the distance between the cell and mRNA distribution centroids by the cell's radius of gyration to normalise for cell size [17]. Consistently, a comparable reduction in PI was observed in HUVECs and HRMECs (Figs. 1C, S1C). Importantly, this result was specific, as mislocalisation of one transcript did not affect the PI of the other (Fig. 1C).

The interaction of trans-acting factors with *RAB13* and *NET1* mRNAs may be disrupted by misloc ASOs, possibly explaining their effects on subcellular mRNA distribution. Recently, the RNA-binding protein CNBP was shown to participate in subcellular mRNA distribution [8]. Hence, we tested CNBP binding to *RAB13* and *NET1*, but only RAB13 misloc ASO led to a reduction, though non-significant, in CNBP binding to the target mRNA (Fig. S2A, B).

Considering that 3′ UTRs are critical for posttranscriptional regulation, ASO binding could potentially alter transcript levels or translation dynamics and thereby confound phenotypic analyses. Thus, we first confirmed that cells treated with ctrl and misloc ASOs contained comparable levels of *RAB13* and *NET1* mRNAs (Figs. 1D, S1D). Next, we carried out polysome fractionation in HUVECs and observed that misloc ASOs induced a mild, but non-significant, shift in the relative transcript abundance from sub-polysome to polysome fractions (Fig. S2C, D). However, this did not result in protein accumulation, as Western blotting showed equivalent amounts of RAB13 and NET1 (Figs. 1E, S1E). Collectively, these data demonstrate that 2′-MOE-PS ASOs targeting specific LEs in *RAB13* and *NET1* are effective and selective tools to manipulate mRNA localisation in endothelial cells.

### Endothelial cell chemotaxis is disrupted by mislocalisation ASOs

**Fig. 2:**
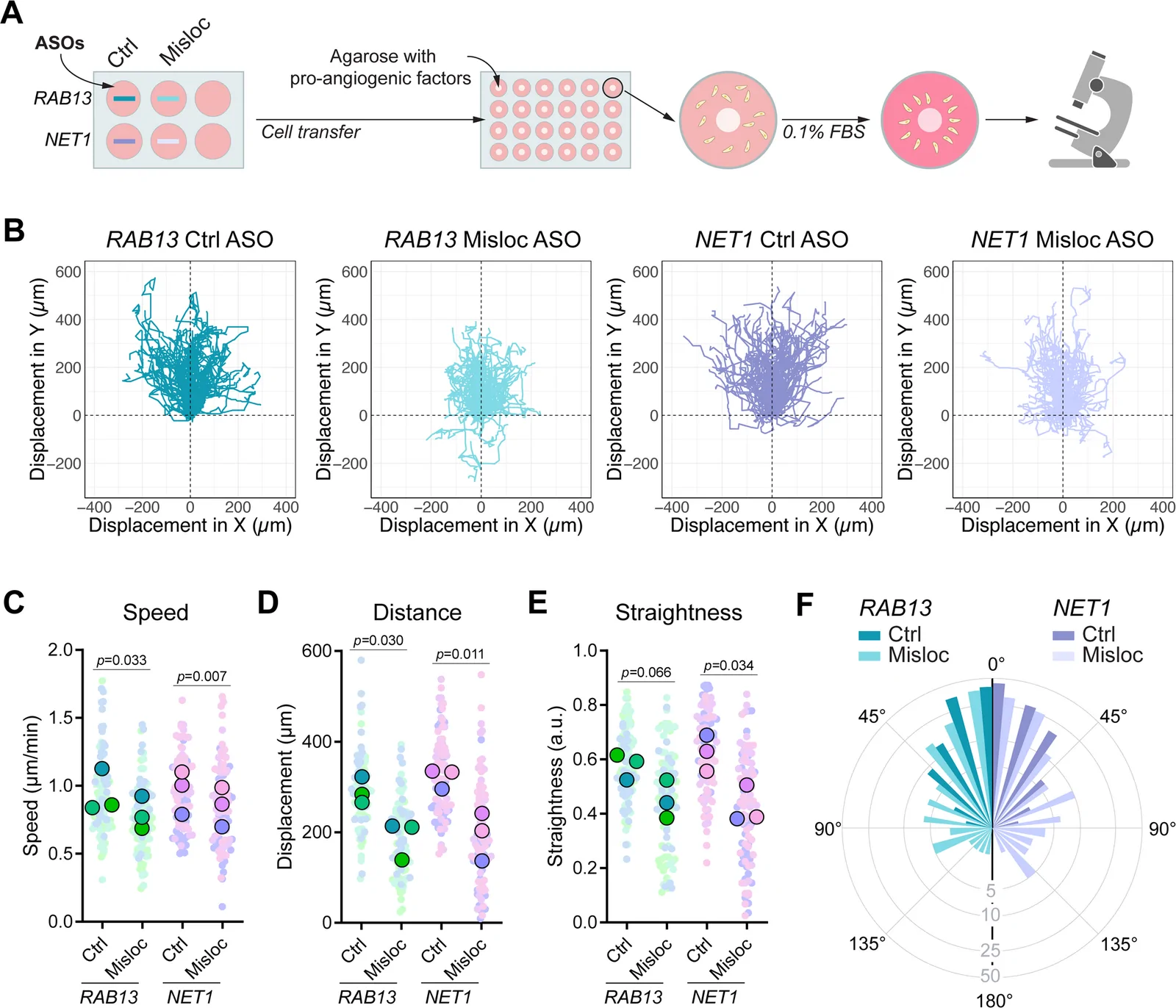
Loss of mRNA polarisation disrupts endothelial cell chemotaxis. **A** Schematic illustrating the strategy to assess migration of ASO-treated HUVECs in the agarose spot assay. **B** Representative track plots showing movement trajectories of individual HUVECs. **C**–**E** Scatter plots quantifying speed (C), total displacement (D) and straightness (E) of cell migration. **F** Rose histograms represent directionality of cell migration relative to the agarose spot; 0° indicates the position of the chemoattractant source. N = 3 biological replicates, 32 cells per replicate; paired t-tests

Chemotactic migration is a critical endothelial cell behaviour during angiogenic sprouting and simple in vitro motility assays allow assessment of cellular responses to chemoattractant gradients [18]. Hence, we employed agarose spot migration assays [19] combined with time-lapse imaging to evaluate the effect of misloc ASOs on chemoattractant-guided endothelial cell motility (Fig. 2A). Compared with cells pre-treated with ctrl ASOs, endothelial cells pre-cultured with either with *RAB13* or *NET1* misloc ASOs displayed a marked reduction in motility towards pro-angiogenic cues over a six-hour imaging period (Fig. 2B). Quantitative analysis of motility parameters revealed that misloc ASOs decreased the speed of cell movement as well as the total distance travelled (Fig. 2C, D). In addition, analysis of net displacement from start to end point demonstrated that misloc ASO-treated cells presented a reduction in linear motion (Fig. 2E). To determine whether orientation of movement towards the chemoattractant source was also impaired, we computed the angle between the net displacement vector of each cell relative to the position of the agarose spot. This analysis showed that misloc ASO-treated cells were significantly less aligned with the chemoattractant source than cells treated with ctrl ASOs (Fig. 2F). Importantly, the deficits in endothelial chemotaxis caused by misloc ASOs are likely attributable to disrupted cell polarity and impaired response to environmental cues, rather than a general loss of cell motility. In chemoattractant-free experiments, time lapse imaging and cell tracking showed no major deficits in endothelial motility following *RAB13* nor *NET1* misloc ASO treatment (Fig. S3A, B). Although mRNA mislocalisation caused a mild reduction in migration speed, total distance and overall directionality were comparable between ctrl or misloc ASO-treated cells (Fig. S3C–F). Together, these findings indicate that disrupting *RAB13* and *NET1* mRNA polarity with ASOs hinders endothelial cell chemotaxis towards pro-angiogenic factors.

### Mislocalisation ASOs inhibit endothelial cell sprouting in vitro

Angiogenic sprouting entails the tight coordination of endothelial cell behaviours in complex environments [20]. We therefore extended our analyses beyond endothelial cell motility to determine whether misloc ASOs also affect vessel formation in in vitro angiogenesis assays. We initially employed the fibrin gel bead assay [21], a simple three-dimensional model that recapitulates early stages of sprouting angiogenesis (Fig. S4A). Using cells pre-treated with a non-targeting (NT) FAM-labelled 2′-MOE-PS ASO, we first confirmed efficient ASO uptake and persistence during sprouting in fibrin gels (Fig. S4B). Endothelial cells were then pre-treated with either *RAB13* or *NET1* ASOs prior to fibrin gel assays. While ctrl ASO cultures exhibited substantial sprouting after 24 h, both *RAB13* and *NET1* misloc ASO-treated cultures showed a marked reduction in angiogenic activity over the same period (Fig. S4C). Quantitative analysis revealed that *RAB13* and *NET1* misloc ASOs significantly reduced the number of capillaries emerging per bead (Fig. S4D), as well as the average length and the number of branched vessels (Fig. S4E,F).

**Fig. 3:**
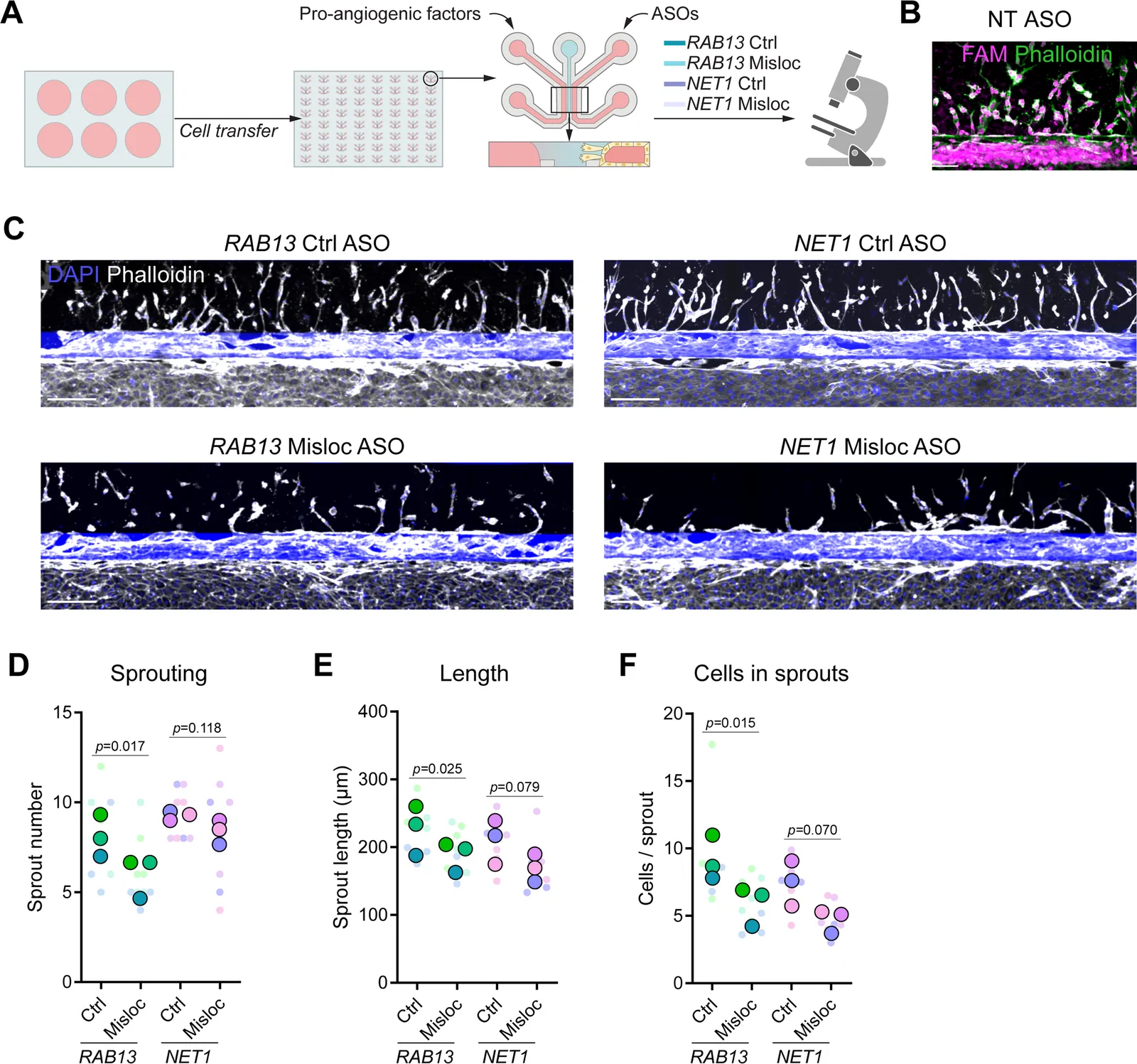
Mislocalisation ASOs hinder sprouting angiogenesis. **A** Schematic illustrating the strategy used to test the angiogenic potential of ASO-treated HUVECs in “vessels-on-a-chip” OrganoPlates. **B** Representative image showing the presence of a non-targeting (NT) FAM-ASOs in angiogenic sprouts. **C** Representative images of endothelial sprouts formed in OrganoPlates. **D**–**F** Scatter plots quantifying, sprout number (D), sprout length (E) and number of cells per sprout (F); n = 3 biological replicates; 3 chips per replicate; paired t-tests. Scale bars: 200 µm

However, reduced sprouting in misloc ASO-treated cultures could reflect impaired cell adhesion to the beads rather than a direct loss of sprouting capacity. To exclude this possibility, we next employed a “vessel-on-a-chip" microfluidic platform, which enables in situ delivery of molecular agents immediately prior to angiogenic induction. In this system, endothelial cells line a chamber to form a perfusable microvessel-like tubule that can be accessed via media inlets for ASO delivery. Sprouting is then induced into an adjacent collagen gel by a gradient of pro-angiogenic factors (Fig. 3A). We first exposed endothelial tubules to a single dose of NT FAM-labelled ASO and confirmed efficient uptake and persistence three days later (Fig. 3B). Following this protocol, we evaluated the anti-angiogenic potential of *RAB13* or *NET1* misloc ASOs. Both reduced endothelial sprouting compared to ctrl ASOs (Fig. 3C); however, *RAB13* misloc ASOs had a substantially stronger effect than their *NET1* counterparts. *RAB13* misloc ASOs markedly supressed vessel invasion into the collagen matrix, yielding fewer and shorter branches with reduced numbers of incorporated cells relative to control cultures (Fig. 3D–F). In contrast, differences in sprouting between *NET1* ctrl and misloc ASOs cultures were observable, but considerably more modest.

In conclusion, our in vitro experiments indicate that *RAB13* and *NET1* misloc ASOs inhibit angiogenesis, although their relative efficacy appears to depend on the distinct characteristics of each assay.

### mRNA mislocalisation ASOs exhibit anti-angiogenic effects in a choroidal sprouting assay

**Fig. 4:**
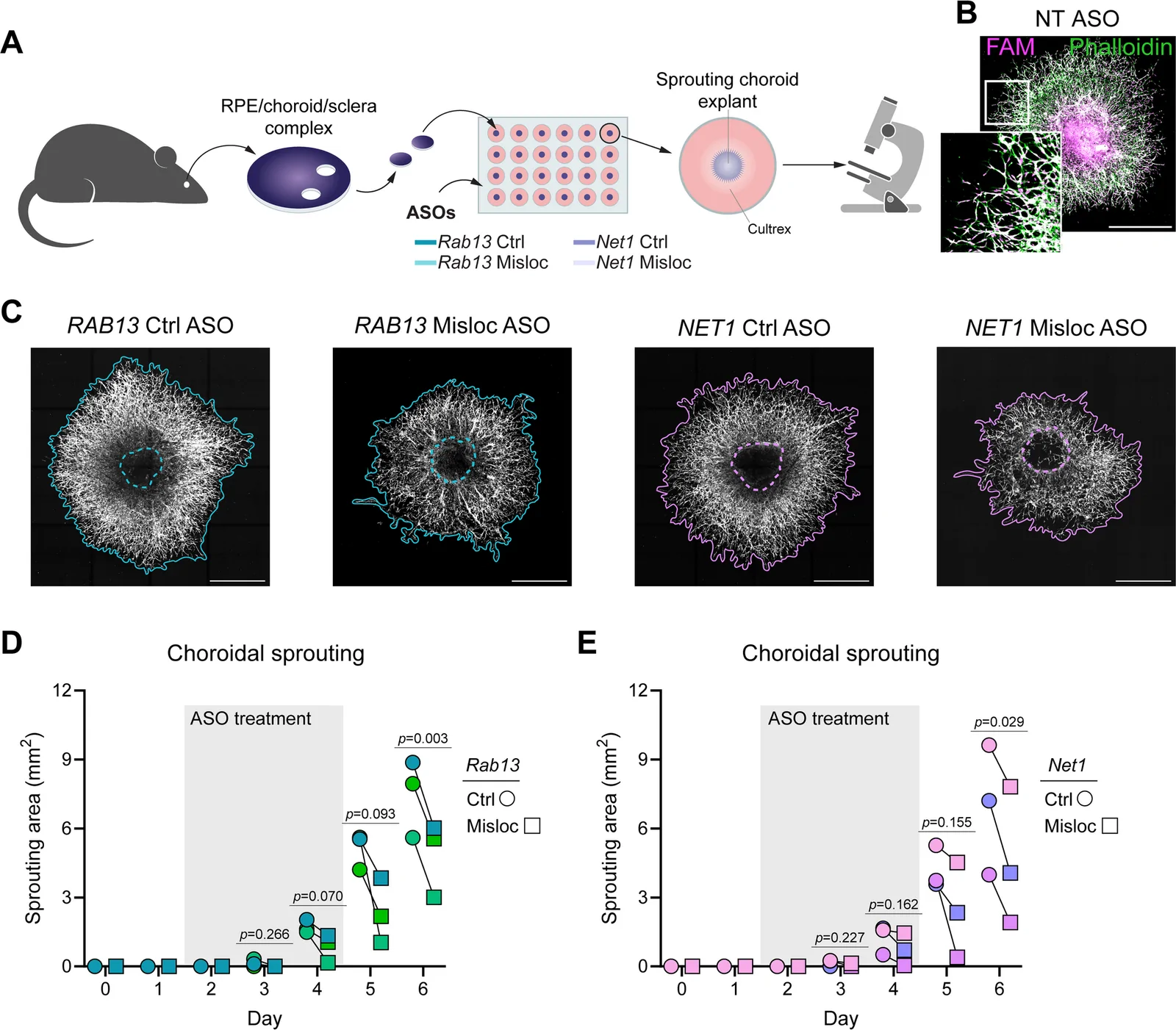
ASOs targeting LEs reduce choroidal explant sprouting. **A** Schematic showing the experimental strategy used to evaluate the effects of ASOs on choroidal explant sprouting. **B** Representative image showing the presence of a non-targeting (NT) FAM-ASO within choroidal sprouts. **C** Representative images of choroidal explants; outer outlines indicate the area of choroidal outgrowth and inner outlines indicate region of the choroid explant. **D**, **E** Plots showing quantification of choroidal sprouting area; n = 3 biological replicates; paired t-tests. Scale bars: 1 mm

Despite the marked effects of misloc ASOs on vessel sprouting, their efficacy may, in part, reflect the simplistic nature of the in vitro models. As described above, readily accessible endothelial cells are typically maintained in monoculture systems that lack the cellular complexity of vascularised tissues. To address this limitation, distinct explant-based assays have been developed to study microvasculature angiogenesis in the presence of other tissue resident cells, including fibroblasts and immune cells [22–25]. One such model uses murine choroid, a vessel-rich ocular layer positioned between the retina and the sclera that provides metabolic support to retinal tissue. Murine choroids can be readily isolated from enucleated eyes and upon dissection into small fragments, embedded in extracellular matrices that support potent outward sprouting. Isolectin B4 (IB4) labelling indicates that sprouts emerging from the explants are endothelial in origin, as virtually all cells within the sprouts are IB4-positive (Fig. S5A). Accordingly, the choroidal sprouting assay is widely regarded as a robust model to investigate regulators of angiogenesis and the influence of non-endothelial cells on vessel formation. We therefore adapted this model to assess the impact of ASO-mediated of mRNA mislocalisation on vessel growth (Fig. 4A). First, NT FAM-labelled 2′-MOE-PS ASOs were added to embedded choroidal fragments and maintained in culture for 72 h. Two days later, NT ASOs were broadly distributed throughout the newly formed vessels, confirming efficient ASO uptake by endothelial cells in this setting (Fig. 4B). To test whether mRNA mislocalisation impairs angiogenesis in choroidal explants, we designed misloc 2′-MOE-PS ASOs targeting LEs within the 3′ UTRs of the murine orthologues *Rab13* and *Net1,* along with corresponding ctrl ASOs directed against 3′ UTR regions lacking reported LEs (Table S1). Exposure of explants to either *Rab13* or *Net1* misloc ASOs resulted in significantly reduced areas of vessel outgrowth compared with ctrl ASO-containing cultures (Figs. 4C, S5B). Time course analysis revealed that effects of misloc ASOs were detectable from the earliest stages of sprouting. Although no sprouts were observed in any condition before day three, explants treated with misloc ASOs displayed a delay in sprout emergence and a sustained reduction in vessel outgrowth. Notably, *Rab13* and *Net1* misloc ASOs produced comparable effects across all time points analysed. By day six, the area occupied by vessels in misloc-treated cultures was significantly reduced (Fig. 4D, E).

Collectively, these ex vivo data demonstrate the anti-angiogenic efficacy of *Rab13* and *Net1* misloc ASOs in a tissue-derived system that better reflects the cellular complexity of vascularised environments.

### Retinal revascularisation is limited by mislocalisation ASOs

**Fig. 5:**
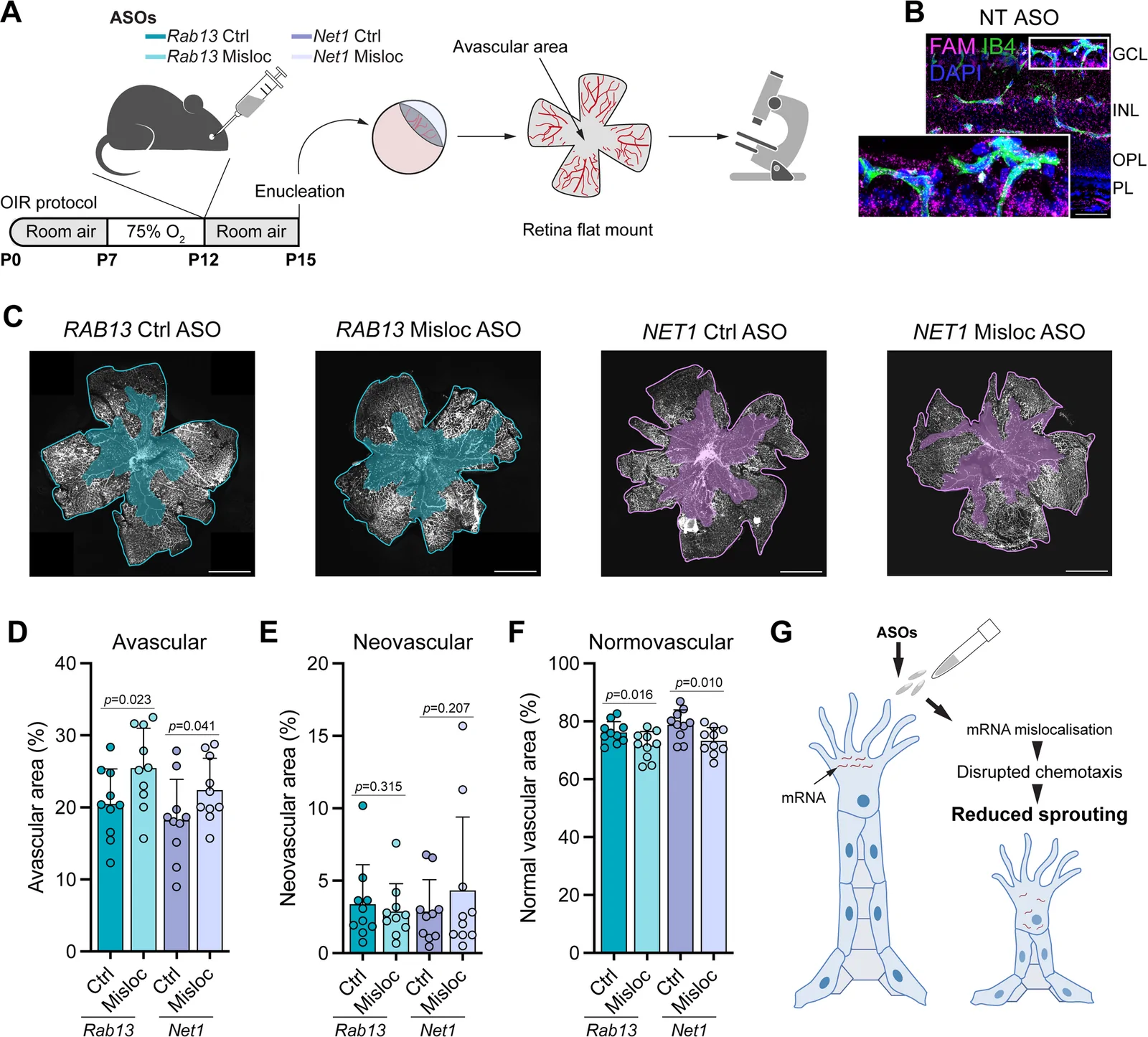
Mislocalisation ASOs inhibit angiogenesis in vivo. **A** Schematic illustrating the experimental strategy used to test the effects of ASOs in the OIR model. **B** Representative confocal image showing the incorporation of a non-targeting (NT) FAM-ASO across distinct layers of the mouse retina and its presence in IB4-labelled blood vessels. GCL, Ganglion Cell Layer; INL, Inner Nuclear Layer; OPL, Outer Plexiform Layer. **C** Representative images of OIR retinas labelled with IB4; coloured overlays indicate avascular areas. **D**–**F** Scatter plots with bars show quantification of avascular (D), neovascular (E) and normally vascularised (F) areas; n = 10 biological replicates; means ± standard deviation; unpaired t-tests. **G** Scheme summarises the inhibitory effects of misloc ASOs on angiogenic sprouting. Scale bars: 50 µm (B) and 1 mm (C)

The mouse retina provides a well-established in vivo system for studying the molecular and cellular mechanisms that govern angiogenesis. A widely used model within this context is oxygen-induced retinopathy (OIR) [26]. In this model, exposure of mouse pups to hyperoxia induces vessel obliteration primarily in the central retina. Subsequent return to normoxia elicits a strong hypoxia-driven neovascular response, including centripetal angiogenic sprouting into previously avascular regions (Fig. 5A). Owing to its reproducibility and the accessibility of the retina for intravitreal delivery, the OIR model provides an ideal platform to evaluate anti-angiogenic strategies. Indeed, antisense-based approaches have previously been applied successfully in this system [27, 28]. To study the efficacy of misloc ASOs in the model, we first examined if ASOs are taken up by native retinal endothelial cells. Intravitreal injection of NT FAM-labelled 2′-MOE-PS ASOs resulted in widespread distribution across multiple retinal layers. Notably, IB4-labelled endothelial cells appeared to show enriched ASO uptake (Figs. 5B, S6A). We therefore proceeded to evaluate vessel growth in mice injected with *Rab13* and *Net1* misloc ASOs following return to normoxia. Compared with control eyes, retinas from misloc ASO-injected eyes exhibited significantly larger vaso-obliterated areas in the central retina, indicating defects in vascular expansion into this region (Fig. 5C). Quantitative analysis revealed that the *Rab13* misloc ASO produced a stronger and more reproducible effect than the *Net1* misloc ASO (Fig. 5D). Retinas exposed to ctrl and misloc ASOs showed comparable levels of *Rab13* and *Net1* (Fig. S6B), indicating that misloc ASOs do not affect the expression of their targets. Likewise, retinal expression of key neovascular modulators, including *Vegfa*, *Kdr* and pro-inflammatory cytokines, remained largely unchanged (Fig. S6C, D). Because body weight is a determinant of neovascularisation in the OIR model, we compared animal weights prior to sacrifice and found no significant differences across experimental groups (Fig. S6E). Overall, this assessment further supports the conclusion that misloc ASOs directly limit vessel growth.

A key feature of OIR is the emergence of pathological vascular tufts, which are clusters of immature and disorganised vessels that protrude into the vitreous [29]. Although tuft formation varied substantially across samples, neither *Rab13* nor *Net1* misloc ASOs produced consistent differences in tuft areas compared with controls (Fig. 5E). This suggests that the reduced proportion of normal vascularised areas in both misloc ASO-treated eyes (Fig. 5F) is primarily due to impaired revascularisation of vaso-obliterated regions, rather than altered pathological tuft formation.

Together, these findings demonstrate that *Rab13* and *Net1* misloc ASOs limit angiogenesis in vivo and underscore the potential of mRNA localisation as a mechanistic target for controlling vessel formation.

## Discussion

As understanding of the molecular and cellular mechanisms underpinning angiogenesis expands, exciting new ways to manipulate vessel growth are paved [20]. We previously showed that restricting subcellular mRNA distribution regulates endothelial cell morphology during angiogenesis. However, those studies involved genetic manipulations that are difficult to apply across experimental models [5]. By employing ASOs, a versatile tool for controlling RNA metabolism, we not only further demonstrate that mRNA localisation is necessary for adequate vessel sprouting, but also propose that this mechanism is targetable to suppress angiogenesis (Fig. 5G). We tested ASOs across models of increasing complexity, which revealed distinct levels of angiogenesis suppression. This could be explained by the nature of the pro-angiogenic environments created to promote vessel sprouting. The supra-physiological concentration of factors used to induce angiogenesis in “vessels-on-a-chip” or the abrupt hypoxic-driven responses in OIR may dampen the contribution of spatially restricted mRNA regulation to vessel sprouting.

Broadly, ASOs targeting *RAB13* and *NET1* LEs display comparable anti-angiogenic properties across models. This observation may reflect convergence between the pathways in which the encoded proteins operate. *RAB13* encodes a member of the Rab family of small GTPases that modulates cytoskeletal remodelling and its activation at the plasma membrane is required for cell migration and invasion [30–32]. RAB13 also facilitates RhoA activity, another small GTPase critically involved in endothelial cell motility [33]. Meanwhile, *NET1* encodes a guanine nucleotide exchange factor for RhoA and thus also promotes cytoskeleton reorganisation [34–37]. Although newly synthesised RAB13 and NET1 proteins do not remain localised near their sites of translation, spatially restricted production facilitates specific co-translational interactions that influence binding partner selection and shape functional outcomes of nascent proteins [9, 10]. Disturbing such processes could underpin the anti-angiogenic effects of misloc ASOs. In addition, our observations suggest that translation efficiency may be slightly enhanced, although whether and how this phenomenon is mechanistically linked with partner interaction and local protein function remain to be understood.

Of note, in angiogenesis models of higher complexity, ASOs targeting *RAB13* LEs promoted slightly stronger responses than those targeting *NET1* LEs. Differences in efficacy may reflect variation in target site accessibility within local RNA secondary structure, which can influence ASO hybridisation. In line with this, in silico analyses of the 3′ UTR suggest that *RAB13* LE is more accessible and requires less energy to unfold than *NET1* LE in both human and mouse transcripts. By contrast, *NET1* LE appears to be embedded within more stable secondary structures, which may hinder efficient ASO binding (Table S5). Although these predictions do not account for the full complexity of RNA structure in vivo, they provide a plausible mechanistic contribution to the observed differences. Such structural features could therefore partially account for the stronger effects observed with ASOs targeting *RAB13*.

Alternatively, differences may arise from the broader roles of RAB13 on multiple pathways relevant to sprouting and thus, *RAB13* mRNA mislocalisation is likely to perturb greater numbers of core molecular components underpinning angiogenesis. At the front of migrating cells, RAB13 controls the trafficking and spatial localisation of RhoA and upstream activators, thereby ensuring precise local RhoA activation required for cytoskeletal contractility at the cell leading edge [33]. In parallel, RAB13 engages the effector MICAL-L2 to remodel the actin cytoskeleton independently of RhoA, stabilising protrusions and coordinating lamellipodia dynamics [30–32]. This dual control means that RAB13 altered activity disrupts both RhoA-dependent inputs and a major actin remodelling pathway, leading to defects in polarity and migration. By contrast, NET1 represents a single RhoA GEF activation route within a broader and potential redundant network of RhoA regulators [38]. Thus, in the context of cell motility behaviour, RAB13 activity has a substantially broader and more dominant impact on RhoA signalling than NET1.

ASOs that mislocalise mRNAs have been reported elsewhere as PMO formulations and were used to demonstrate that targeting LEs blocks the assembly of transport-competent complexes involving CNBP [8]. Although yet to be fully determined, 2′-MOE-PS misloc ASOs may disrupt binding of CNBP to *RAB13* but not to *NET1* mRNA. Targeting *NET1* LE could prevent other non-identified RNA-binding proteins from distributing *NET1* without altering CNBP binding. This points to a broader principle that LE function, and ASO-mediated disruption, operate through transcript-specific mechanisms. In fact, GA-rich LEs have been identified in other protrusion-enriched mRNAs beyond *RAB13* and *NET1* [5]. For example, an LE in *TRAK2* mRNA is responsible for polarised distribution of the transcript in endothelial cells [7]. However, given that loss of this LE enhances endothelial cell motility, ASOs targeting it would not be expected to have effects similar to those of *RAB13* and *NET1* misloc ASOs.

Undesirable vessel formation is a major pathological factor associated with vasculopathies underpinning diverse diseases [20]. Among the many strategies developed to curb angiogenesis, ASOs are emerging as promising therapeutic agents, and some have progressed to late-stage clinical trials. These compounds incorporate diverse ribose modifications and mostly target pro-angiogenic factors with broad biological functions [39]. Importantly, ASO chemistry is a key determinant of efficacy as different modifications can produce varying levels of toxicity. We used ASOs containing 2′-MOE-PS modifications that have generally been reported to promote enhanced stability, distribution, and efficacy in modulating RNA metabolism [40]. Previous studies comparing common chemistries for retinal applications such as 2′-OMe-PS, 2′-MOE-PS and PMO, showed 2′-MOE-PS to be the most promising for intravitreal delivery. While 2′-MOE- and 2′-OMe-based chemistries exhibited broadly similar profiles, 2′-MOE often showed greater efficacy across multiple targets. In contrast, PMOs have yielded mixed results: modified PMOs were associated with significant in vivo toxicity, whereas unmodified PMOs remained non-toxic but displayed poor retinal uptake [41]. Such observations suggest that the 2′-MOE-PS ASOs we used to mislocalise with *Rab13* and *Net1* are efficacious in vivo. Whether they can be equally applied to limit angiogenesis in other tissues remains to be investigated.

Our in vivo studies employing the OIR model revealed that misloc ASO reduced revascularisation of vaso-obliterated regions, with no consistent effects on tuft formation. These findings suggest that targeting mRNA localisation may primarily inhibit regenerative rather than pathological angiogenesis in this model. Revascularisation depends on directional endothelial cell migration led by polarised tip cells toward the ischemic regions, while tufts are formed by growth in a less organised manner into the vitreous [42]. Noteworthy, loss of the polarity determinant ATP6AP2 in endothelial cells has been shown to reduce regenerative angiogenesis in the OIR model, with no apparent effects om tuft formation [43]. In contrast, the core polarity regulators NCK1 and NCK2 are broadly required for both types of vessel growth [44]. The lack of effects of misloc ASOs on neovascular tufts therefore suggests that perturbing mRNA localisation does not disrupt global aspects of tip cell polarity, but instead interferes with fine-tuned regulation of polarity and response to guidance cues. In light of these findings, misloc ASOs are likely to exhibit context-dependent translational potential determined by the relative reliance on distinct mechanisms underpinning vessel growth. While they may have limited utility in ischaemic retinopathies, in which effective therapies involve concomitant suppression of pathological neovascularisation and improvement of physiological angiogenesis, misloc ASOs could have stronger benefits in settings where inhibition of undesired vessel growth is the primary objective. Future studies entailing the efficacy of misloc ASOs in models beyond OIR will be important to further define potential therapeutic implications.

Most ASO-based strategies modify protein expression levels through degradation of target mRNAs or through steric blockade of translation or splicing factors [45]. In cases where the targeted protein has pleiotropic roles, such modulation is likely to produce effects across multiple cellular pathways. Additionally, the methods for ASO delivery lack tissue specificity [46], hence, altered protein expression in undesired cell types may result in detrimental side effects. For this reason, ASOs modulating spatial control of gene expression without resorting to overall changes in protein levels could overcome such problems and offer unprecedented benefits. By altering subcellular mRNA distribution underpinning cell polarity, this strategy may be mechanistically effective only in particular cellular states, such as endothelial cells undergoing angiogenesis. Although further studies providing more detailed evaluation of safety and therapeutic potential will be required, mRNA localisation presents a promising mechanistic target to dissect healthy and pathological angiogenesis.

## Materials and methods

### Endothelial cell culture and ASO treatments

Primary HUVECs (PromoCell) were cultured on gelatin-coated culture dishes in complete Endothelial Cell Growth Medium 2 (ECGM2; PromoCell), supplemented with 50 μg/mL gentamicin (Merck) and 50 ng/mL amphotericin B (Merck). Primary HRMECs (Innoprot) were cultured on fibronectin-coated culture dishes in complete Endothelial Cell Medium (Innoprot). Cells were maintained at 37 °C in a humidified incubator with 5% CO₂ and used at passages 3–6. In all in vitro assays, 2′-MOE-PS ASOs (IDT; Table S1) were added to endothelial cells at a final concentration of 5 μM for gymnotic uptake in complete media and standard culture conditions. Experimental replicates were independently obtained using distinct cell stocks.

### RNA isolation and RT-qPCR

Total RNA from cells and retina lysates was extracted using the RNeasy Mini Plus Kit (Qiagen), and equal amounts of RNA were transcribed into cDNA using the High-Capacity cDNA Reverse Transcription Kit (Thermo Fisher Scientific), following manufacturers’ instructions. RT-qPCR was carried out with 1 µL cDNA, 0.25 μM gene‐specific primers (Merck; Table S2) and 1 × SYBR Green Master Mix (Roche) using a Roche LightCycler 480 (Roche). Relative gene expression levels normalised to *GAPDH* (cells) or *Actb* (retinas) were determined using the 2^−ΔΔCt^ method.

### Western blotting

Protein extracts were prepared with RIPA buffer supplemented with a protease inhibitor cocktail (10%, Sigma-Aldrich). Protein concentrations were quantified using the BCA Protein Assay Kit (Thermo Fisher Scientific), following the manufacturer’s instructions. Total protein lysate (10 or 20 µg) were resolved on 10 or 12% SDS–polyacrylamide gels and transferred onto either nitrocellulose membranes (RAB13 blotting) or PVDF membranes (NET1 blotting). Membranes were blocked for 1 hour in 5% bovine serum albumin (Sigma-Aldrich) in TBS containing 0.1% Tween-20 (TBST), followed by incubation with primary antibodies (Table S3) overnight at 4 °C. After washes with TBST, membranes were incubated with HRP-conjugated secondary antibodies (Table S3) for 1 hour at room temperature. After washes with TBST and a wash with TBS, signal was developed using Clarity Western ECL Substrate (Bio-Rad) and detected with a Syngene G:Box imaging system (Syngene, Cambridge, UK).

### smFISH

Quasar 570-conjugated smFISH probes targeting human *RAB13* and *NET1* mRNAs (Table S4; LGC Biosearch Technologies) were designed using the Stellaris Probe Designer (LGC Biosearch Technologies). Cells were seeded on coated coverslips and incubated with ASOs for 48 hours. Cells were then fixed with 4% formaldehyde (Thermo Fischer Scientific) for 5 minutes at room temperature, followed by permeabilization in 70% ethanol for 1 hour at room temperature or overnight at 4 °C. Cells were washed in wash buffer (2 × SSC, 10% formamide) for 5 minutes and incubated in hybridization buffer (10% dextran sulfate, 2 × SSC, 10% formamide) containing 125 nM smFISH probe overnight at 37 °C. Post-hybridization, samples were washed twice in wash buffer for 30 minutes at 37 °C and once in 2× SSC containing 1 µg/ml DAPI (Merck) for 5 minutes at room temperature. Coverslips were mounted on microscopy slides using ProLong Gold Antifade Mountant (Thermo Fisher Scientific). smFISH imaging was undertaken on Nikon Eclipse Ti-E (Nikon) microscope equipped with a 100x/1.45/NA/CFI60 Plan Apochromat Lambda oil objective lens and a Neo 5.5 sCMOS ANDOR camera. Z-stack images were acquired with a step size of 0.2 μm and deconvolved using NIS-A Elements software (Nikon). Processed smFISH images were analysed to quantify mRNA polarisation using the Park Lab’s Localization Analysis plugin for ImageJ [17, 47].

### Cell motility assay

HUVECs treated with ASOs for 24 hours were transferred in complete ECGM2 into 24-well plates (5,000 cells per well), either empty or containing 5 µL 0.5% agarose (Gibco) spots supplemented with 250 nM S1P (Merck), 20 ng/mL PMA (Merck), 37.5 ng/mL rhFGF-b (Thermo Fisher Scientific), 37.5 ng/mL rhMCP-1 (Merck), 37.5 ng/mL rhHGF (Merck), and 37.5 ng/mL rhVEGF-165 (RayBiotech). Cultures without agarose spots were imaged 24 hours later, while spot-containing counterparts were changed to M199 (Gibco) with 0.1% FBS for 3 hours. Brightfield live-cell imaging was conducted using a Nikon Eclipse Ti-E (Nikon) microscope equipped with a 10×/0.13/ NA/CFI60 Plan Fluor DLL objective lens and a Neo 5.5 sCMOS ANDOR camera. Cells were maintained at 37 °C in 5% CO₂ and were imaged every 5 minutes over a 6-hour period, capturing 4 fields per chemoattractant-free well or 4 quadrants of agarose spot. Individual cells were manually tracked in ImageJ and resulting trajectories were subsequently analysed using CelltrackR [48] in RStudio: Integrated Development Environment for R (Posit Software).

### Fibrin gel bead assay

The assay was performed for analysis of sprouting angiogenesis as previously described [21]. Briefly, HUVECs treated with ASOs for 24 hour were harvested and incubated with Cytodex 3 microcarrier beads (Merck) in complete ECGM2 for 4 hours at 37 °C, with gentle agitation every 20 minutes to ensure uniform coating. HUVEC-coated beads were transferred to T25 flasks and incubated overnight to promote cell attachment. Beads were then washed 3 times with complete ECGM2 and embedded in a mixture of fibrin gel (Merck), aprotinin (Merck) and thrombin (Merck) within glass-bottom 96-well plates. After 16 hours, cultures were washed with PBS and fixed with 4% formaldehyde for 15 minutes at room temperature. The cultures were permeabilised in PBS containing 0.5% Triton X-100 for 5 minutes at 4 °C, followed by PBS washes. Nuclei were stained with 1 µg/ml DAPI, and F-actin was labelled with ActinGreen Ready Probe (Thermo Fisher Scientific) for 30 minutes at room temperature. After 3 additional PBS washes, samples were maintained in PBS for imaging. Beads were imaged using Leica STELLARIS 5 laser scanning confocal microscope (Leica Microsystems) with a 20x/0.75NA/HC/PL/APO/CS2 objective lens, controlled with Leica Application Suite X (LAS X) software (Leica Microsystems). Z-stack images were acquired with a 2 μm step size.

### Microfluidic “vessels-on-a-chip” cultures

Microfluidic cell cultures were performed using OrganoPlates 3-lane 64 (MIMETAS), according to manufacturer guidelines. Briefly, 2 µL of 4 mg/mL neutralized collagen I (R&D Systems) were loaded into gel channels and polymerized at 37 °C for 15 minutes. Next, 2 µL of 10,000 cells/µL suspension were seeded into right perfusion inlet, followed by the addition of 50 µL of media. The plates were then placed in the incubator on a stand for 4 hours to allow cell attachment to collagen. Then, 50 µL complete ECGM2 was added to right perfusion outlets and left perfusion inlets and outlets of each chip. Microvessels were allowed to form for 2 days, and ASOs were then added to right perfusion inlet and outlet in media. On day 3, angiogenic cocktail composed of 125 nM S1P, 10 ng/mL PMA, 18.75 ng/mL rhFGF-b, 18.75 ng/mL rhMCP-1, 18.75 ng/mL rhHGF, and 18.75 ng/mL human VEGF-165 were added to the left perfusion inlet and outlet to induce sprouting from microvessels. Cultures were fixed with 4% formaldehyde on day 5 and processed for staining according to manufacturer guidelines. Nuclei were stained with 1 µg/ml DAPI, and F-actin was labelled with ActinGreen Ready Probe for 30 minutes at room temperature. After 3 additional PBS washes, samples were maintained in PBS for imaging. Leica SP8 laser scanning confocal microscope (Leica Microsystems) was used to image the chips with a 10x/0.4/NA/HC/PL/FLUOTAR objective lens, controlled with (LAS X) software. The tilescan feature was used to capture 3 adjacent tiles, which were merged, and Z-stack images were acquired with a 4.3 μm step size.

### UV-RNA immunoprecipitation (UV-RIP) and polysome fractionation

UV-RIP was performed as described in Smith et al. [49]. For polysome fractionation, HUVECs (80–90% confluence) were pretreated with 100 µg/ml cycloheximide (Sigma) for 5 minutes and then washed twice with ice-cold PBS containing 100 µg/ml cycloheximide. Ice-cold PBS containing 100 µg/ml cycloheximide was added to the cells, which were then dislodged from the plates by scraping and collected by centrifugation at 1,200 rpm for 5 minutes at 4 °C. The supernatant was discarded, and the cell pellets were then lysed in 500 µl hypotonic buffer (5 mM Tris–HCl pH 7.5, 2.5 mM MgCl2, 1.5 mM KCl, 1× HALT PIC EDTA-free (Thermo Fisher Scientific), 100 U RNAseOUT (Thermo Fisher Scientific), 100 μg/ml cycloheximide, 2 mM dithiothreitol, 0.5% v/w Triton X-100 and 0.5% v/w sodium deoxycholate). Lysates were cleared through centrifugation at max speed for 5 minutes at 4 °C, and the total RNA concentration of each sample was measured at 254 nm using a NanoDrop 2000 instrument (Thermo Fisher Scientific). 100 µg RNA were loaded and separated on 14 mL of a 10–50% sucrose gradient by ultracentrifugation at 36,000 rpm for 2 hours at 4 °C using a SW40Ti rotor in an Optima L-80XP ultracentrifuge (Beckman Coulter). Absorbance at 254 nm was measured from lower to higher sucrose gradients using an ISCO gradient fractionation system, and the optical density at 254 nm was continuously recorded with a Foxy JR Fractionator (Teledyne ISCO). Fractions 1–8 (sub-polysomal) were pooled together, and fractions 9–16 (polysomal) were pooled together. Each pool was spiked with 5 ng/µL Luciferase Control RNA (Promega). RNA was then isolated using TRIzol and chloroform, and precipitated with 1 vol. of isopropanol, 0.1 vol. of sodium acetate, and 1 µL of GlycoBlue Coprecipitant (Thermo Fisher Scientific) overnight at − 80 °C. The samples were centrifuged at maximum speed, the pellets washed with 70% ethanol and resuspended in RNAse-free water. RT-qPCR was conducted as described in the RNA isolation and RT-qPCR methods section. Ct values were normalised to Luciferase controls in polysome fractionation and fold changes were determined using the 2^−ΔΔCt^ method.

### Animals

Mice were housed in standard microisolator cages in the Biological Services Unit at Queen’s University of Belfast and provided food and water ad libitum. Numbers of mice used for each experiment are indicated in the figure legends. All animal maintenance and experiments adhered to the UK Home Office Animals (Scientific Procedures) Act 1986 and were approved by the Animal Welfare Ethical Review Body (AWERB) of Queen’s University Belfast. The experiments also conformed with the Association for Research in Vision and Ophthalmology (ARVO) Statement for the Use of Animals in Ophthalmic and Vision Research.

### Mouse choroidal ex vivo sprouting assay

Fresh eyes were collected from 3-month-old mice and placed in ice-cold 1 × PBS supplemented with 1% penicillin–streptomycin (Thermo Fisher Scientific). Choroidal dissections were performed in the same solution under sterile conditions, leaving the choroid-sclera complex intact. Choroids were flattened by 4 radial incisions and then biopsy punches were collected from the periphery using a 1 mm tissue punch (Medisave). The punches were transferred to pre-warmed complete ECGM2, placed in 24-well plates, and media was removed using a pipette. 30 µL droplets of Cultrex Reduced Growth Factor Basement Membrane Extract (BME), Type 2, Pathclear (R&D Systems) was gently pipetted over each explant, ensuring no bubble formation and maintaining a circular shape. Next, explants were incubated for 45 minutes at 37 °C to allow Cultrex solidification and complete ECGM2 was added to each well before transferring to incubator. Next day, ASOs were added to media at 5 μM and ECGM2 was replaced 72 hours later. For bright field daily imaging, a Leica DMi8 microscope (Leica Microsystems) was used with a 10x/0.32/PH1/HC/PL/FLUOTAR objective lens, controlled with LASX software. The tilescan feature was used to capture 20 adjacent tiles, which were subsequently merged. On day 6, explants were washed with cold PBS and fixed with 4% formaldehyde for 10 minutes at room temperature. Nuclei were stained with 1 µg/ml DAPI, and F-actin was labelled with ActinGreen Ready Probe for 24 hours at 4 °C. Vessel labelling was carried out by permeabilization with 10% Triton X-100 for 10 minutes at room temperature followed by incubation with biotin-conjugated IB4 (1:200) from *Bandeiraea simplicifolia* (Sigma-Aldrich) for 24 hours at 4 °C. After PBS washes, explants were incubated with streptavidin conjugated to Alexa Fluor 488 (1:200) (Invitrogen) for 24 hours at 4 °C. After 3 additional PBS washes, samples were maintained in PBS for imaging. Leica SP8 laser scanning confocal microscope was used to image labelled choroidal sprouts with a 10x/0.4/NA/HC/PL/FLUOTAR objective lens, controlled with LAS X software. The tilescan feature was used to capture 16 adjacent tiles, which were merged, and Z-stack images were acquired with a 4.3 μm step size.

### OIR model and retina flat mount preparation

Ischemic retinopathy was induced in C57BL/6 mice according to the original protocol by Smith et al. [26]. Briefly, P7 mice and their nursing mothers were exposed to 75% oxygen (OxyCycler; Biospherix Ltd.) for 5 days to induce vasodegeneration and atrophy of the central retinal capillary beds. Mice were removed from the oxygen chamber at P12 and returned to normal room air. At this stage, mice were intravitreally injected with 0.5 µL of a PBS solution containing 5 μg/µL ASOs using a 34-G bevelled needle (Nanofil; World Precision Instruments) under general anaesthesia. Pups were sacrificed at P15 for enucleation of eyes to assess endothelial proliferation and pathological sprouting caused by hypoxia in the avascular retina.

Eyes were fixed in 4% formaldehyde for 24 hours at 4 °C and retinal wholemounts were prepared, stained, and flatmounted. Briefly, retinas were washed with PBS and blocked for 3 hours in buffer containing 1% bovine serum albumin and 0.5% Triton-X 100 in PBS. Vessel labelling was carried out by incubation with biotin conjugated IB4 (1:200) prepared in blocking buffer for 24 hours at 4 °C. After PBS washes, retinas were incubated with streptavidin conjugated to Alexa Fluor 488 (1:200) (Invitrogen) prepared in blocking buffer for 24 hours at 4 °C. The retinas were then washed extensively with PBS and mounted onto microscopic slides using ProLong Gold Antifade Mountant.

For the staining of retina cryosections, eyes were fixed in 4% formaldehyde for 1 hour at room temperature post-enucleation. The eyes were then washed with PBS and cryoprotected in 10%, 20% and 30% graded sucrose solutions (Sigma-Aldrich, USA), before being embedded in optimal cutting temperature compound and cryo-sectioned using Leica CM 1900 cryostat (Leica Microsystems, Milton Keynes, UK) at 14 μm thickness. IB4 labelling was performed following above protocol and nuclei were stained with 1 µg/ml DAPI before mounting using ProLong Gold Antifade mountant. Leica SP8 laser scanning confocal microscope was used to image the retinal flatmounts with a 10x/0.4/NA/HC/PL/FLUOTAR objective lens, controlled with LAS X software. The tilescan feature was used to capture 20–25 adjacent tiles which were subsequently stitched together. The retinal cryosections were imaged with 40x/1.25/NA/HCX/PL/APO/PH3/oil objective lens and Z-stack images were acquired with a 2 μm step size, controlled with LAS X software. Vascular, avascular and neovascular areas were quantified as a percentage of the total area of each retina following standard procedures [50].

### Statistical analysis and RNA structure prediction

All data were analysed using GraphPad Prism software. The Shapiro–Wilk test was performed to evaluate the normality of data distribution (*p* > 0.05). A Student’s t-test was used to compare differences between each control and respective experimental group. For comparisons involving more than two groups, one-way ANOVA was performed, followed by Dunnett’s multiple-comparisons test. Statistical significance is reported as exact *p *values. Rose histograms were generated in RStudio. Prediction of RNA secondary structures were carried out using the ViennaRNA Package [51].

## Supplementary Information

Below is the link to the electronic supplementary material.

Supplementary Material 1

## Data Availability

All data supporting the findings of this study are available within the article and its Supplementary Information. The code used in this study is available from the corresponding author upon request.
