# Supplementary material for: Antisense oligonucleotide-mediated disruption of mRNA localisation inhibits angiogenesis
Source: Angiogenesis. 2026 Aug 20;29(4):61. doi: 10.1007/s10456-026-10087-z (PMC13493432; doi:10.1007/s10456-026-10087-z)
Supplement: Supplementary file 1 [file 10456_2026_10087_MOESM1_ESM.pdf]

SUPPLEMENTARY MATERIAL

Antisense oligonucleotide-mediated disruption of mRNA localisation inhibits angiogenesis

Supplementary figures (Fig. S1-S6)

Supplementary tables (Table S1-S5)

References

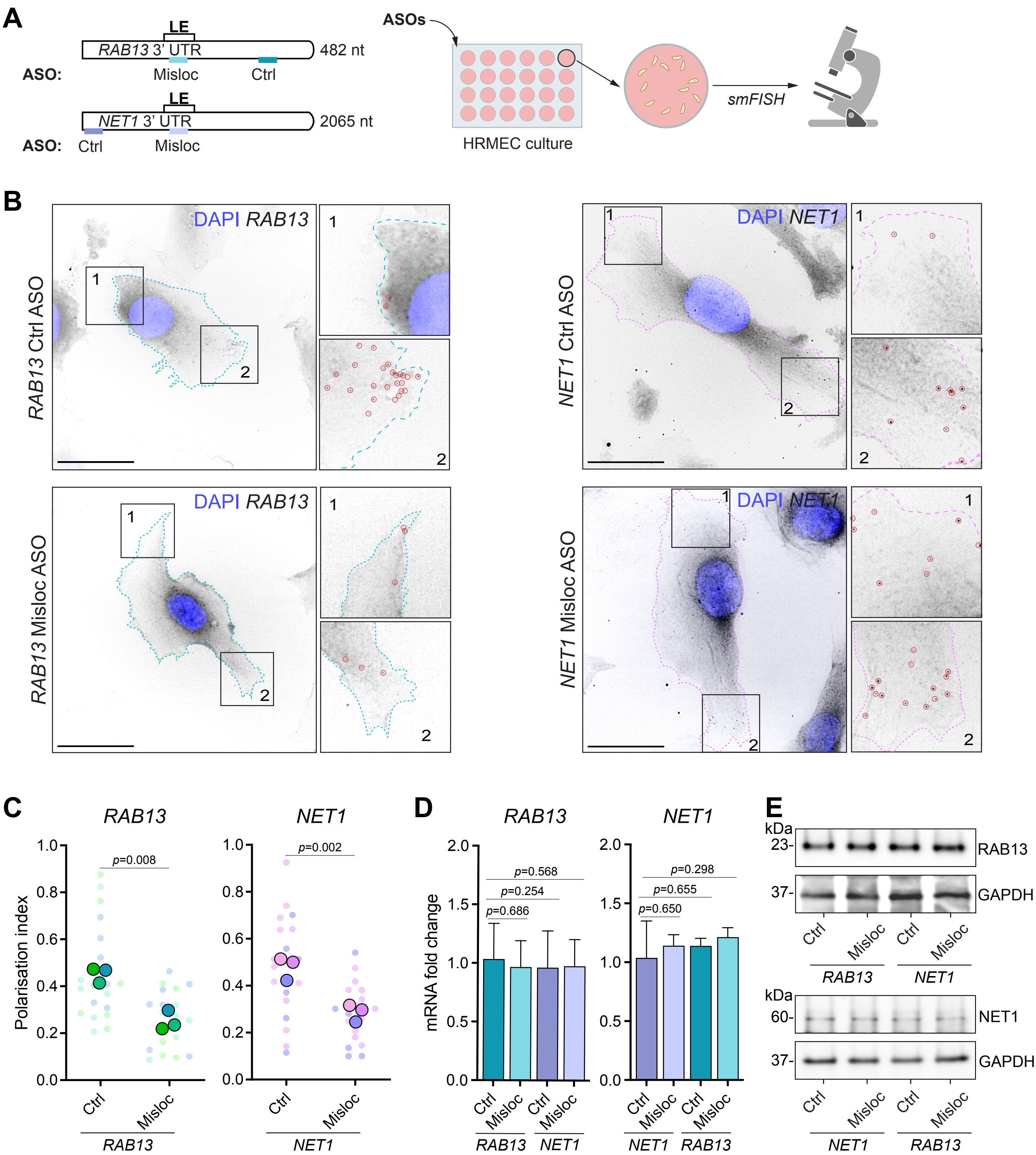

Figure S1 – 2'-MOE ASOs targeting LEs disrupt mRNA localisation in HRMECs. (A) Diagram depicts 3' UTR positions targeted by *RAB13* and *NET1* ASOs (left), and strategy to assess their mRNA mislocalisation potential in HRMECs via smFISH (right). **(B)** Representative *RAB13* and *NET1* smFISH images; insets illustrate the relative abundance of smFISH puncta at each cell pole; scale bars: 50 µm. **(C)** Scatter plots show the polarisation index of *RAB13* and *NET1* mRNAs; n=3 biological replicates, ≥ 6 cells per replicate; one-way ANOVA followed by Dunnett's multiple comparison test. **(D)** Bar graphs represent RT-qPCR quantification of *RAB13* and *NET1* mRNA in HRMECs; n=3 biological replicates; means ± standard deviation; one-way ANOVA followed by Dunnett's multiple comparison test. **(E)** Representative blots of RAB13 and NET1 levels in HRMECs.

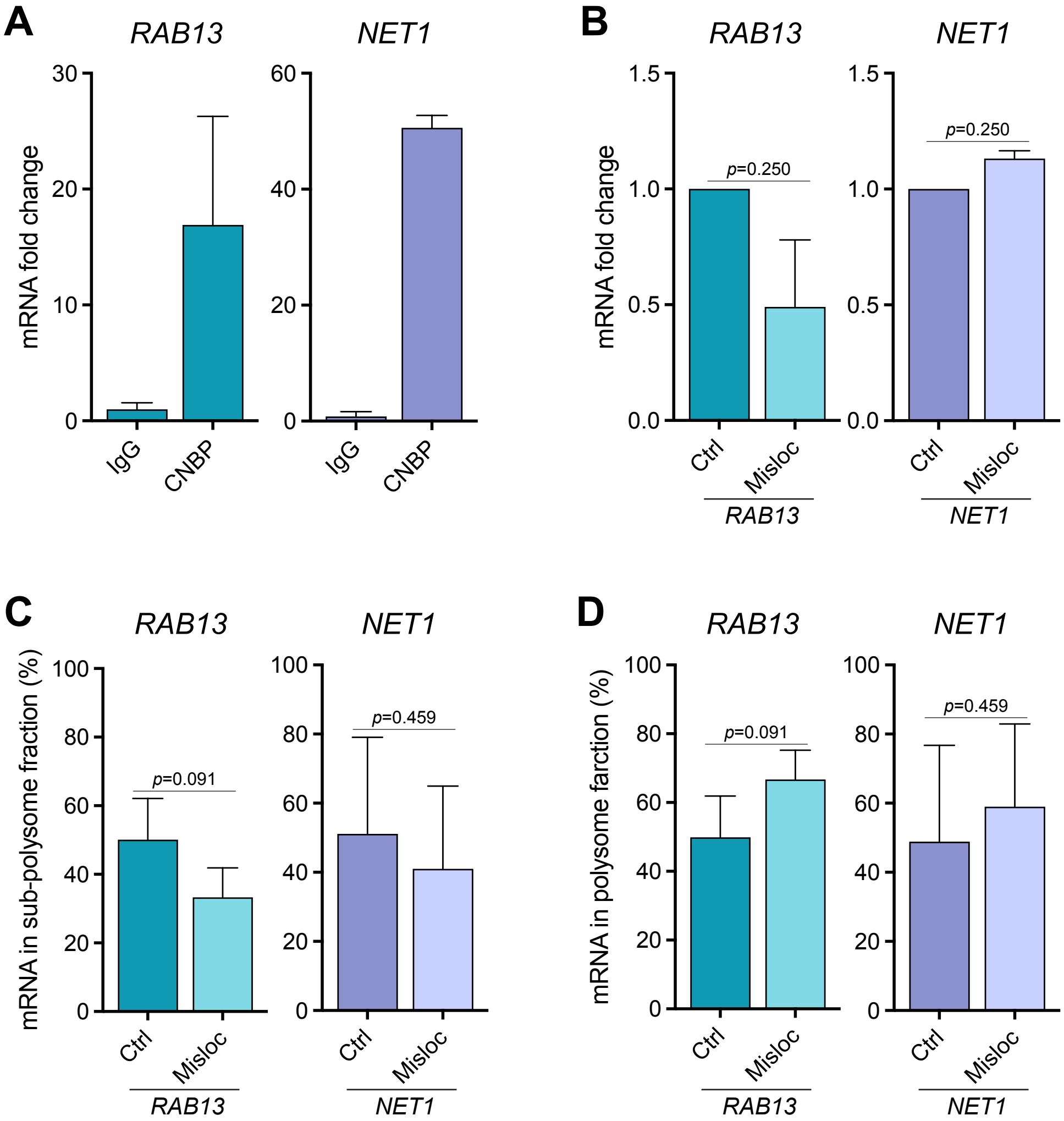

Figure S2 – Effects of 2'-MOE-PS ASOs targeting LEs on mRNA and protein. (A) Bar graphs represent RT-qPCR quantification of *RAB13* and *NET1* mRNA pulled-down in test CNBP UV-RIP; n=2 biological replicates; means ± standard deviation. **(B)** Bar graphs represent RT-qPCR quantification of *RAB13* and *NET1* mRNA bound by CNBP; n=3 biological replicates; means ± standard deviation; Wilcoxon signed-rank test. **(C, D)** Bar graphs represent RT-qPCR quantification of *RAB13* and *NET1* mRNA present in **(C)** sub-polysome and **(D)** polysome fractions relative to total; n=3 biological replicates; means ± standard deviation; paired t-tests.

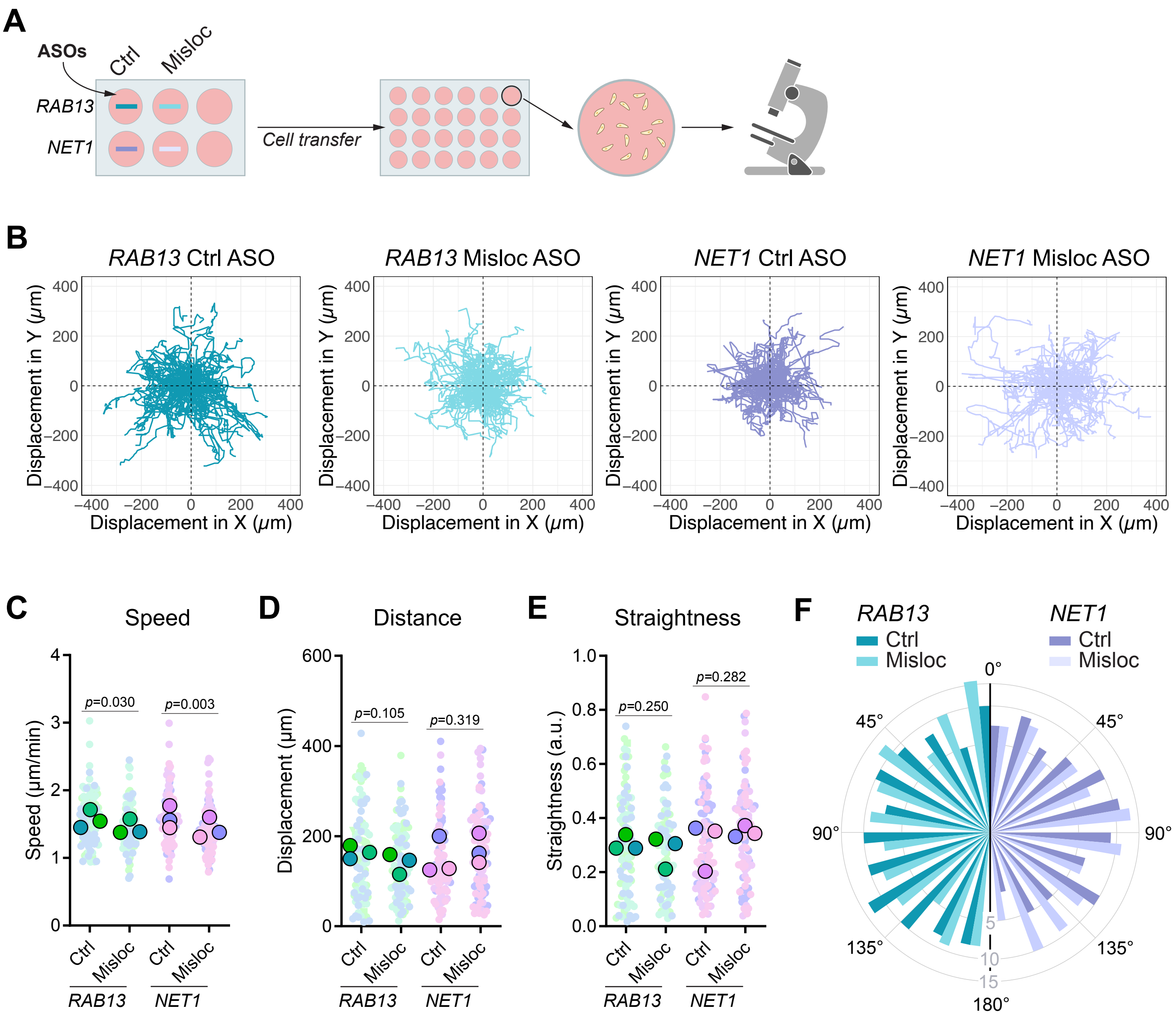

Figure S3 – Loss of mRNA polarisation does not disrupt endothelial cell movement. (A) Schematic illustrating the strategy to assess migration of ASO-treated HUVECs. **(B)** Representative track plots showing movement trajectories of individual HUVECs. **(C-E)** Scatter plots quantifying **(C)** speed **(D)** total displacement and **(E)** straightness of cell migration. **(F)** Rose histograms represent directionality of cell migration. N=3 biological replicates, 32 cells per replicate; paired t-tests.

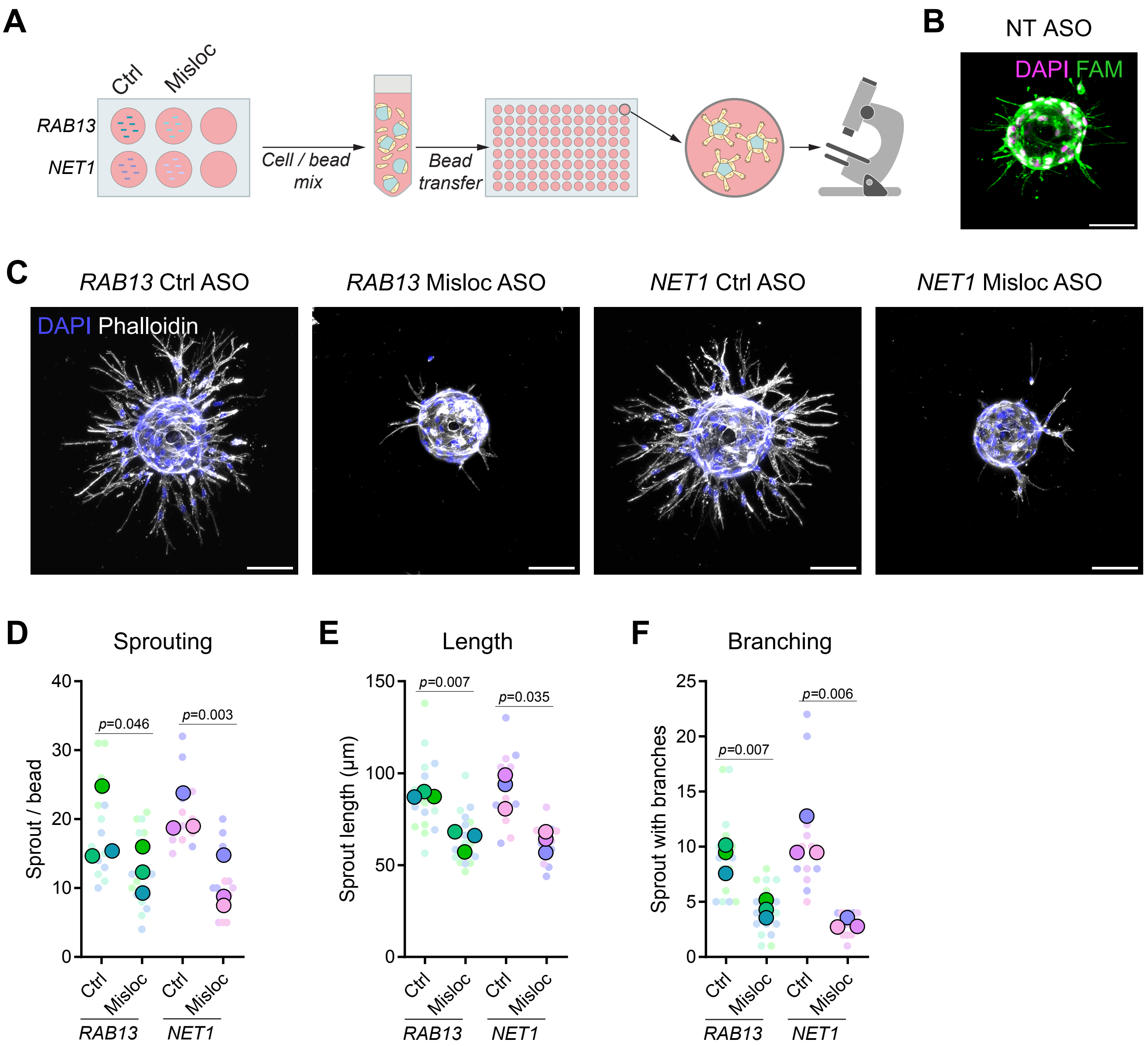

Figure S4 – Mislocalisation ASOs hinder endothelial cell sprouting. (A) Schematic illustrating the strategy used to test the angiogenic potential of ASO-treated HUVECs in fibrin gel bead assays. **(B)** Representative image showing the presence of a non-targeting (NT) FAM-ASOs in angiogenic sprouts. **(C)** Representative images of endothelial sprouts formed in fibrin gel bead assays. **(D-F)** Scatter plots quantifying **(D)** sprout number, **(E)** sprout length and **(F)** sprouts with branches; n=3 biological replicates; 5 beads per replicate; paired t-tests. Scale bars: 100 µm.

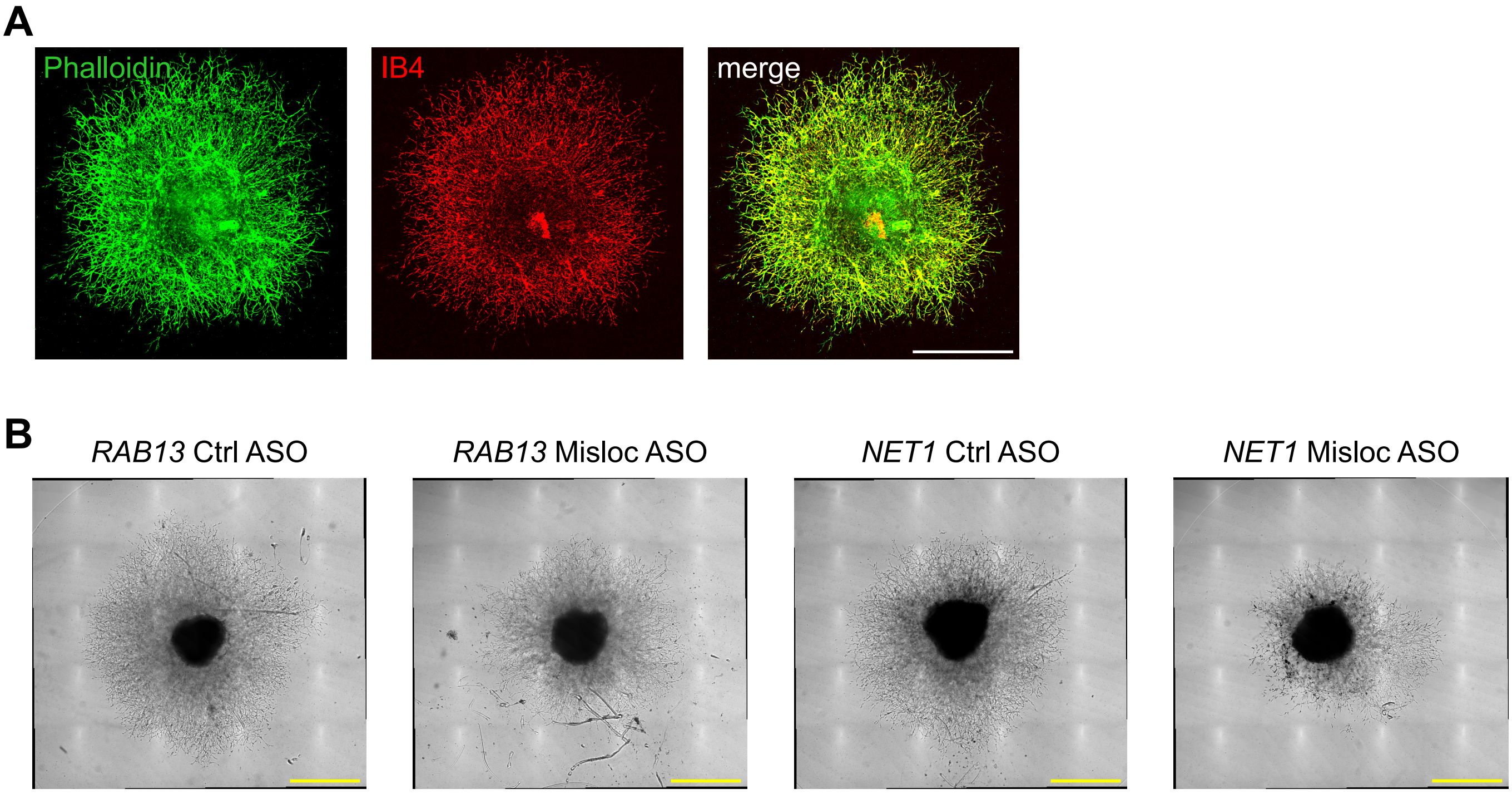

Figure S5 – Endothelial cells in choroidal explants uptake 2'-MOE ASOs. (A) Representative image demonstrates that choroidal sprouts are composed of IB4-labelled endothelial cells. **(B)** Representative bright field images of choroidal explants (related to Figure 4C). Scale bars: 1 mm.

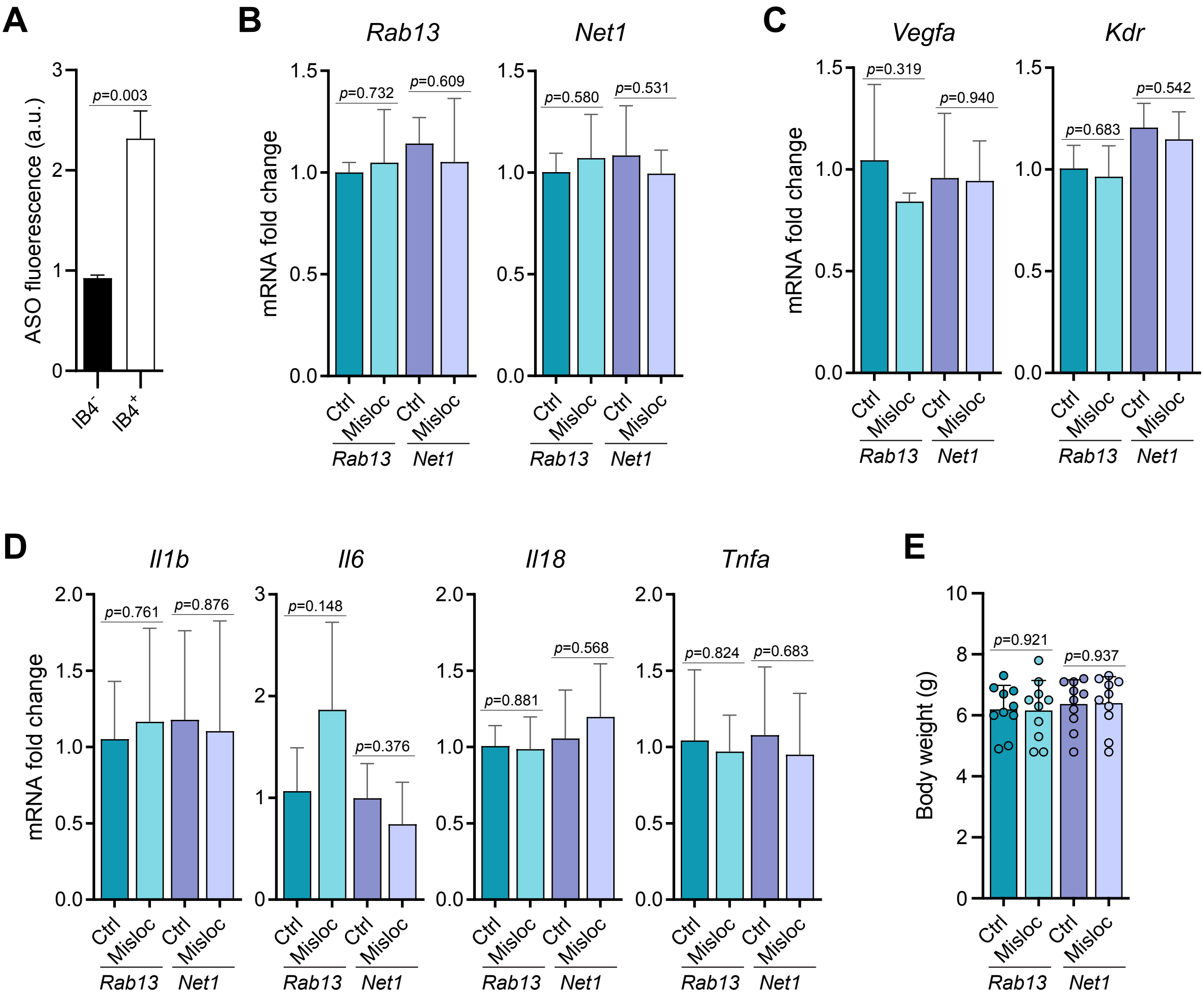

Figure S6 – Analysis of ASO intravitreal injections in OIR animals. (A) Fluorescence from FAM-labelled ASO measured within regions of OIR retinal sections and normalised to total FAM fluorescence (related to Figure 5B); n=4 sections; means ± standard deviation; paired t-test. **(B-D)** Bar graphs represent RT-qPCR quantification of indicated mRNAs in retinas; n=4-5 biological replicates; means ± standard deviation; unpaired t-tests. **(E)** Scatter plots with bars show the quantification of animal body weights (related to Figure 5); n=10 animals; means ± standard deviation; unpaired t-tests.

Table S1 – Sequences of human and mouse targeted 2'-MOE-PS ASOs. *3'-FAM conjugated.

**ASO Target**	**Sequence (5'-3')**	**Source**
RAB13 Control	GACAGAACAGGAGCAAATTCCCTAG	[1]
RAB13 Misloc	TCTTTCACTTCCTCAATTCATTCCT	[1]
NET1 Control	TATAAAGTAACCCTATGATGCTCCC	
NET1 Misloc	TCCCTCTTGCATTTCAGACAACACT	[2]
Rab13 Control	ACTAACAAAGACCTTGTAGAGTGAG	[1]
Rab13 Misloc	TTCACATCCTTGGTTCCTCCCCCTC	[1]
Net1 Control	ACTAAACTATGGACTTTTCTTATCA	[3]
Net1 Misloc	TCCCTTCCCTATCCCAGACTCCTG	[3]
Non-Targeting*	CCTCTTACCTCAGTTACAATTTATA	

Table S2 – Sequences of primers used for RT-qPCRs.

**Name of Gene**	**Forward primer (5'-3')**	**Reverse primer (5'-3')**
GAPDH	TCAAGGCTGAGAACGGGAAG	CGCCCCACTTGATTTTGGAG
RAB13	AAGCCTACGACCACCTCTTC	TCCACAGTGCGGATCTTGAA
NET1	GCACATGATGAGACTGGAGGT	GTGACACGAGCCAGAGGT
Actb	GATTACTGCTCTGGCTCCTAG	GACCATCGTACTCCTGCTTG
Il18	CACAGCCAGTCCTCTTACTTC	TCGTTGACAAAAGACAGCCT
Il6	TCCTTAGCCACTCCTTCTGT	AGCCAGAGTCCTTCAGAGA
Il1b	CTCTTGTTGATGTGCTGCTG	GACCTGTTCTTTGAAGTTGACG
Kdr	AGAAAGCCTTGATCTCGCCT	CCTCCACGTGTCTCCATTCT
Net1	GTCAGAAGAGGAACTCACGCAC	CCATCAGGCTTGGTTGCTTCTC
Rab13	AACAAGTGTGACATGGAGGC	TGGATTTGGCACTCGTCTCA
Tnfa	TCTTTGAGATCCATGCCGTTG	AGACCCTCACACTCAGATCA
Vegfa	CTGCTGTAACGATGAAGCCCTG	GCTGTAGGAAGCTCATCTCTCC
Firefly Luciferase	GAGCACGGAAAGACGATGACGG	GGCCTTTATGAGGATCTCTCTG

Table S3 – List of primary and secondary antibodies.

**Antibody**	**Dilution**	**Supplier**	**Catalogue no.**
RAB13	1:500	Atlas	HPA003996
NET1	1:1000	Abcam	ab113202
GAPDH	1:10000	Invitrogen	AM4300
Goat anti–mouse HRP	1:3000	Bio-rad	170-6516
Goat anti–rabbit HRP	1:3000	Bio-rad	170-6515
CNBP	1:5000	Proteintech	67109-1-Ig

Table S4 – Stellaris probe sequences used for smFISH.

***RAB13* (5'-3')**	***NET1* (5'-3')**
CAGGCCAGGAAGAAGTTTTC	GCCAGGTGTTAAGAATGTGA
TGGACGGTTGGCAAACAGAG	TCTCTGCGTTTTCTTTTCAA
CAGCAACTTGAAGAGGTGGT	CCAGAGGTCGAACTCTTTTA
ATGATCAGACAAGTCTTGCC	AATTTGCCAAGGACGTGACA
GTTGTTGAAGTTGTCCTCTG	GGTCACCACGAAGGGTAAAT
TTCCGATGGTGGAGATGTAA	CCTGCTAGAAAACTTCTGGG
CCACAGTGCGGATCTTGAAA	AGAGACTCCTTCATGGTGAT
CCAGACTTGTAGTTTGATCT	ATTAAATCCTGTTCACCTCG
ATTGTCTTGAACCGCTCTTG	TCTTGCAAGTTTGAGATCCT
TCCACGGTAGTAGGCAGTAG	AACATGGGGTCATGATAGGC
ATACTAGGATAATGCCCATG	ATATGTGTGAGTTCCTCTTC
GATTTCTCATCCGTGATGTC	GTAAGAGTCCAGATCACCAA
CCAGTTCTGAATATTCTCGA	GCTCACGAGAATGTGACCAA
CATTCTCCTTGATGCTTTTC	AGCTGGTTACTACAGTAACC
CTCCATGTCACATTTGTTCC	GATCCTGTTTCTTTTGATCA
AAATCGGATTCCATGCTCTC	TTCGACTGAAGGGAGACTCG
CTGGATTTAGCACTAGTTTC	GGGATATCTAGGAAACTCCA
AAAAGCCTCATCCACATTCA	GGTATTTGACTAGGCGACTT
CTCCTGACTTGAGCAAGATG	AACATCAGGGTGCTCTTTTG
AGTTTTCAGGTCAGTACTGG	TCAATATAGCATCCTCCAGA
ACTTGTTGGTGTTCTTCTTG	CGGATTCACCTTTCTTCAAG
AGGCAAGAAAGGGTCCTCAG	CCGAGTCAGAACCAAGATGT
TACCTATGTGACCCTCCAAG	GGTAAACCTGGTAAGAGTGC
AACCCAGGTAAGGTCTGAAG	CTGAGTTACTGAAAGCTCCT
TTTACATTTATGTTTGCCCT	CTTGTGGAACACGTCATTGG
GGACCCTAAAACCTGATCTA	CGCTCGAATACAGTTGAACC
GAGCAAATTCCCTAGTGTAG	GTGTCTGGTGTGTCTTTAAG
AGACCATGACAAGTGACAGA	TACACCAAAGTCTCTTTCCG
TGCAAATGGTGGCCTTTAAT	CAGTTAACACACAGAGCCTT
	AAATACCAGGCTTACCATGT
	TGGCAGAATTCCAGCATTTG
	ACATTCCATGTAGGTTTTGG
	GATAAAAACCTCCTCAGGCT
	AACAGTATGGCTTGGTTTCA
	ATGGGTCTTTACCCTGAAAT
	CCTCTTGCATTTCAGACAAC
	CCCCTTTCAAAGATGATGAT
	CACCATTATAATCGGGCTTT
	ACCAGGCAAGAAACCTTCAG
	CTTTTATGAGGCTTACCACT
	CAAGACTTACCTACCTTTCG
	TGTGTCTGATTACACCATGT
	TACTACCCTTACTATCCTAT
	ATTAATCAGGATCCTTTGCC
	CTGGTGTTGCAGATCACAAT
	GCTGATCAAGGTAACTCTGG
	TTTTACCTTTGAGGGAACCA
	TTACTCAGATTCTGGGTCTT

Table S5 – Predicted structural and energetic properties in 3'UTR target sites [4] (P unpaired: probability the site is structurally unpaired, ΔG open: energy required to expose the target region).

**Target**	**P unpaired**	**ΔG open (kcal/mol)**
Human *RAB13*	0.10	1.41
Human *NET1*	0.0012	4.14
Mouse *Rab13*	0.0002	5.18
Mouse *Net1*	0.00006	6.01

References

1. Moissoglu K, Stueland M, Gasparski AN, Wang T, Jenkins LM, Hastings ML, et al. RNA localization and co-translational interactions control RAB13 GTPase function and cell migration. EMBO J. 2020;39(21):e104958.

2. Gasparski AN, Moissoglu K, Pallikkuth S, Meydan S, Guydosh NR, Mili S. mRNA location and translation rate determine protein targeting to dual destinations. Mol Cell. 2023;83(15):2726-38 e9.

3. Mason DE, Madsen TD, Gasparski AN, Kong D, Jiwnani N, Lechler T, et al. Control of epithelial tissue organization by mRNA localization. Nat Commun. 2025;16(1):5216.

4. Lorenz R, Bernhart SH, Honer Zu Siederdissen C, Tafer H, Flamm C, Stadler PF, et al. ViennaRNA Package 2.0. Algorithms Mol Biol. 2011;6:26.
